# Helical growth during the phototropic response, avoidance response, and in stiff mutants of *Phycomyces blakesleeanus*

**DOI:** 10.1038/s41598-021-83254-5

**Published:** 2021-02-11

**Authors:** Joseph K. E. Ortega, Revathi P. Mohan, Cindy M. Munoz, Shankar Lalitha Sridhar, Franck J. Vernerey

**Affiliations:** 1 Mechanical Engineering, University of Colorado Denver, Denver, USA; 2 Mechanical Engineering, University of Colorado Boulder, Boulder, USA

**Keywords:** Model fungi, Cell growth, Biopolymers in vivo, Membrane structure and assembly, Light responses

## Abstract

The sporangiophores of *Phycomyces blakesleeanus* have been used as a model system to study sensory transduction, helical growth, and to establish *global* biophysical equations for expansive growth of walled cells. More recently, *local* statistical biophysical models of the cell wall are being constructed to better understand the molecular underpinnings of helical growth and its behavior during the many growth responses of the sporangiophores to sensory stimuli. Previous experimental and theoretical findings guide the development of these local models. Future development requires an investigation of explicit and implicit assumptions made in the prior research. Here, experiments are conducted to test three assumptions made in prior research, that (a) elongation rate, (b) rotation rate, and (c) helical growth steepness, *R*, of the sporangiophore remain constant during the phototropic response (bending toward unilateral light) and the avoidance response (bending away from solid barriers). The experimental results reveal that all three assumptions are incorrect for the phototropic response and probably incorrect for the avoidance response but the results are less conclusive. Generally, the experimental results indicate that the elongation and rotation rates increase during these responses, as does *R*, indicating that the helical growth steepness become flatter. The implications of these findings on prior research, the “fibril reorientation and slippage” hypothesis, global biophysical equations, and local statistical biophysical models are discussed.

## Introduction

### Growth and development of the sporangiophores of *Phycomyces blakesleeanus*

**Figure 1:**
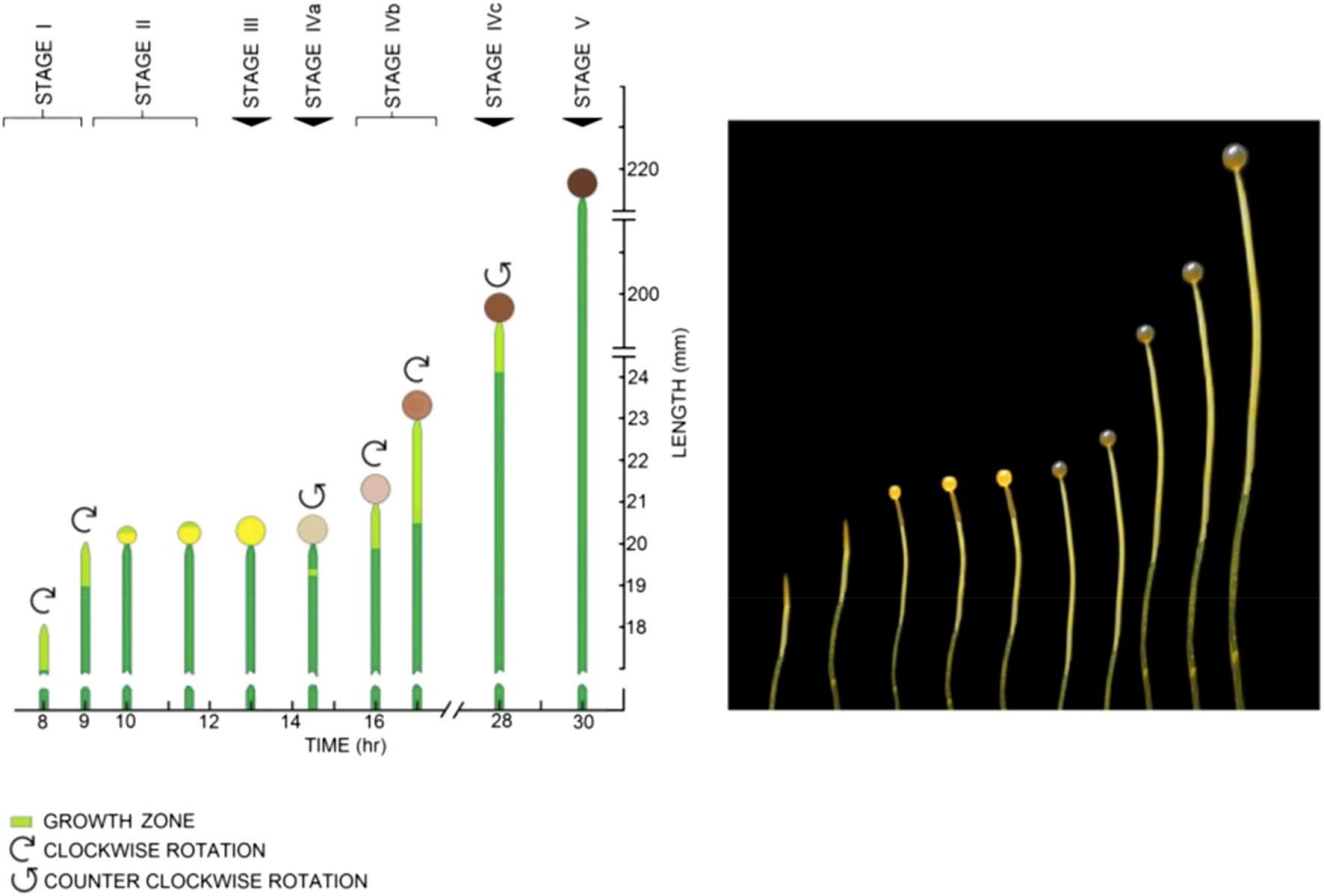
The schematic illustration (left) and corresponding photograph (right) show the developmental stages of the sporangiophore of *P. blakesleeanus*. Sporangiophore development is divided into five stages (stages I, II, III, IV and V), and stage IV is further divided into three sub-stages (IVa, IVb and IVc). The sporangiophores exhibit elongation and spherical growth (volumetric or expansive growth) in a region termed the “growth zone” which is colored light green in the schematic illustration. The non-growing stalk is colored dark green. The growth zone is located at the apical tip of stage I (tip growth) and stage II (spherical growth) and adjacent to the sporangium (intercalary growth) in stage IV. The stage I sporangiophore is a pointed cell that grows longitudinally at the apical tip in the growth zone that is 1–2 mm in length. Clockwise rotation (when viewed from above) and elongation growth occur concurrently during stage I, producing left-handed helical growth. Stage II begins with spherical growth at the apical tip and does not exhibit elongation or rotation growth. Spherical growth continues until it a sporangium is formed (~ 0.5 mm in diameter) and then it stops. This is stage III, a period of spore formation inside the sporangium. During stage III, there is no visible expansive growth. Stage IVa begins with elongation growth concurrent with counter-clockwise rotation growth (right-handed helical growth) in a short growth zone located approximately 0.6 mm below the sporangium. The length of the growth zone, elongation growth rate, and rotation growth rate increase in magnitude as the right-handed helical growth continues for approximately one hour. Then, the rotation rate gradually decreases to zero, and clockwise rotation begins without interruption of elongation growth. Stage IVb begins with the initiation of left-handed helical growth. Stage IVb sporangiophores exhibit nearly constant growth zone lengths (~ 2.5 mm), elongation growth rates (~ 35 μm/min) and rotation growth rates (~ 12 degrees/min) for many hours. Stage IVc is initiated by counter-clockwise rotation and right-handed helical growth. Stage V does not exhibit visible expansive growth. These large cylindrical single-celled sporangiophores are approximately 150 μm in diameter and can grow in length to ten or more centimeters. [Schematic and photograph are taken from Ortega et al.^5^ and reproduced here with permission].

Wild type sporangiophores of *Phycomyces blakesleeanus* are large cylindrical single-celled hyphae that grow vertically from a mycelium. They undergo five stages of development (stages I, II, III, IV and V), and stage IV is further divided into three sub-stages (IVa, IVb and IVc)^1–3^, see Fig. 1. The stage I and stage IVb sporangiophore exhibit left-handed helical growth in a region of the stalk termed the “growth zone”. In Fig. 1, the growth zone is colored as light green in the schematic illustration. The dark green represents the non-growing stalk. During these two stages the cell wall exhibits both elongation and clockwise rotation (when viewed from above) along the length of its growth zone, i.e. left-handed helical growth. The growth zone is adjacent to the spherical sporangium in stage IVb (intercalary growth). A particle placed on the surface of the sporangium will trace out a left-handed helix in time and space. The elongation rate and rotation rate measured at the sporangium reflect the sum of the respective rates of the incremental regions of wall along the length of the growth zone.

Stage IVb sporangiophores are often used for experiments because they exhibit nearly constant growth zone lengths (~ 2.5 mm), elongation growth rates^4^ (~ 35 μm min^−1^) and rotation growth rates (~ 12 degrees min^−1^) for many hours. A ratio *R* (*R* = rotation rate ÷ elongation rate) can be used to measure of the steepness of the helix traced out in time and space by a particle on the surface of the growth zone or on the sporangium. When *R* is large the helix is relatively flat and when *R* is small the helix is steeper. It was implicitly presumed that *R* is approximately constant for stage IVb sporangiophores.

### Sensory responses

The large single-celled sporangiophores of *P. blakesleeanus* have been used as a model system for studies in sensory transduction because of their growth responses to many sensory stimuli^2,3,6,7^. The sporangiophore can detect gravitational acceleration, ethylene, mechanical stretch, gases, temperature, wind, light intensity, spatially asymmetric distribution of light, the presence of solid objects and changes in turgor pressure. Stage IVb sporangiophores respond to these stimuli with symmetric and asymmetric changes in elongation growth rate, and are termed “growth responses” and “tropic responses”, respectively. For example, the light growth response is an increase in elongation rate that begins approximately four minutes after an increase in light intensity^8^. Experimental evidence demonstrates that the increase in elongation rate is transient, i.e. it returns to its original elongation rate within ten to fifteen minutes. It is shown that this occurs because the cell’s photoreceptors “adapt” to the higher light intensity^9^. Another growth response is the pressure response^10^, a transient decrease in elongation rate that occurs immediately after an increase in turgor pressure exceeding 0.02 MPa. This response appears to be related to the “stretch” response reported by Dennison and Roth^11^.

Stage IVb sporangiophores’ responses to asymmetric distribution of light (phototropic response) and to the presence of a solid barrier (avoidance response) are two examples of tropic responses. The phototropic response can be described as follows. When a stage IVb sporangiophore is subjected to two identical light sources on opposite sides and equal distance away, the sporangiophore will grow vertically between the two. If one of the light sources is turned off, producing a unilateral light stimulus, the sporangiophore will grow (bend) toward the remaining light source after a latency period of approximately four minutes. This is the phototropic response^12,13^. It was postulated that the cross-section of the transparent cylindrical stalk of the sporangiophore acts like a lens, focusing the light on the distal side of the stalk and increasing the light intensity on the distal side. Interestingly, the phototropic response is not transient, i.e. it will continue indefinitely providing the unilateral light stimulus remains. In other words, the cell does not adapt to the unilateral light stimulus. The bending that occurs during the phototropic response was quantitatively explained by differential growth that occurs on the proximal and distal sides of the cylindrical stalk of the sporangiophores. Castle^14^ calculated the differential growth that is required to bend the sporangiophore stalk during the phototropic response by assuming that the proximal wall decreases in elongation rate and the distal wall increases in elongation rate, so that the cell’s overall elongation rate was constant. That the phototropic response does not adapt, and can continue indefinitely, was explained by the rotation of the cell wall in the growth zone during bending. Dennison and Foster^15^ postulate that the rotation of the cell wall during helical growth continuously introduces photoreceptors (postulated to be attached to the plasma membrane, thus they rotate with the cell wall) into the higher intensity of the focused light on the distal side of the stalk. In this way it is postulated that the rotation converts a spatial stimulus into a temporal stimulus that prevents adaptation to the unilateral light stimulus. Experimental results support this hypothesis^15^. It was implicitly assumed that the rotation rate was constant but different along the length of the growth zone.

When a stage IVb sporangiophore that is growing between two identical light sources on opposite sides and equal distance away is subjected to a barrier stimulus, the sporangiophore will detect its presence and grow away after a latency period of one to three minutes. This is the avoidance response^6^. Similar to the phototropic response, the avoidance response does not adapt, i.e. the sporangiophore will continue to bend away from the barrier as long as the barrier remains within one millimeter of the growth zone. Gamow and Bottger^16^ reported experimental evidence demonstrating that rotation of the stalk during helical growth is responsible for the lack of adaptation. It is not known how the sporangiophore detects the presence of the barrier and many studies have been conducted in an attempt to elucidate the intermediate stimulus^17^. A significant amount of research has been conducted to study the phototropic and avoidance responses^2,3^, so it is surprising that the behavior of the elongation rate, rotation rate, and *R* has not been studied during these two tropic responses. It was generally assumed that elongation rate, rotation rate, and *R* remain constant during tropic responses^14,15^. More recently, two mutants (C149 and C216) have been obtained whose sporangiophores exhibit very diminished phototropic and avoidance responses^18–20^. These mutants have been termed “stiff” mutants. A recent study has shown that the elasticity of these mutant sporangiophores is essentially the same as that of wild type sporangiophores^21^. Thus the term “stiff” is not an accurate characterization of these mutants and should probably be termed “viscous” mutants. However to retain continuity of terminology we will continue to call them stiff mutants.

### Helical growth

**Figure 2:**
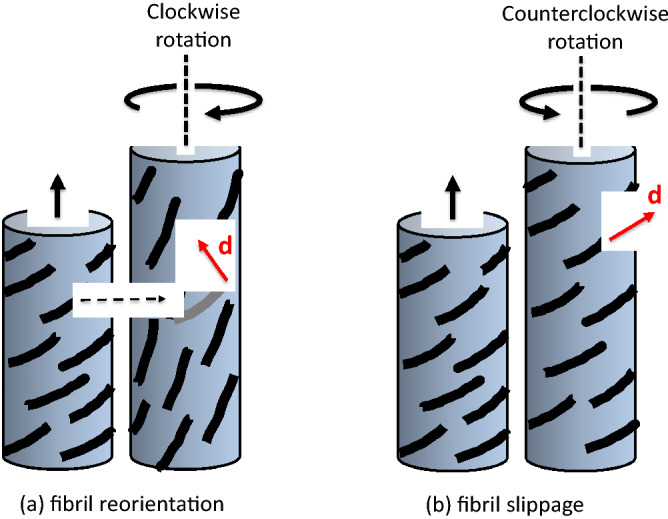
Schematic illustrations of the postulated (**a**) fibril reorientation and (**b**) fibril slippage mechanisms. (**a**) In the fibril reorientation mechanism it is postulated that microfibrils within the cell wall reorient toward the longitudinal axis when the wall undergoes plastic deformation in the longitudinal direction during elongation growth. If the microfibrils are predominately oriented in the direction shown (right-handed helix), a displacement vector, **d**, will be produced by the fibril reorientation. The displacement vector adds a clockwise rotation to the elongation and produces left-handed helical growth. (**b**) In certain situations, it is postulated that the microfibrils slide passed each other along their direction of orientation, i.e. fibril slippage. In this case, the displacement vector, **d**, adds a counter-clockwise rotation to the elongation and produces right-handed helical growth.

Single-celled fungi^22^ and algae^23^ exhibit helical growth. Early attempts to describe and explain helical growth have inspired many experimental investigations and hypotheses^24,27^. Generally these studies and hypotheses have focused on the relationship between the architecture and anisotropic mechanical properties of the primary wall. It is noteworthy that most of these early experimental studies were conducted with the sporangiophores of *P. blakesleeanus*^27^. Subsequent experimental research produced new results concerning the distribution of rotation and elongation rates along the length of the growth zone^28,31^. This experimental research inspired the proposal of a “fibril reorientation” hypothesis (Fig. 2a) that was used to explain the complex helical growth observed along the growth zone during steady growth^30^. Also, a “fibril slippage” hypothesis (Fig. 2b) was proposed^32^ to explain the helical growth reversal during development^1^.

Importantly, the fibril reorientation hypothesis was able to explain the reversal in rotation that occurs during bulging of piloboloid mutant (*pil*-mutant) sporangiophores (an increase in radial expansion that forms a bulge in the sporangiophore stalk) and predicts exactly when the reversal in rotation occurs^33^. In prior research it was implicitly presumed that *R* was constant and independent of the magnitude of steady elongation rate^28–31^. However, subsequent research conducted to verify this assumption demonstrated that *R* changes with the magnitude of the steady elongation growth rate^34^. It was found that *R* is largest for slow growing sporangiophores and decreases almost exponentially to a smaller value (but not zero) for faster growing sporangiophores. Furthermore, it was found that *R* increases during the pressure response. These findings were used to modify the fibril reorientation hypothesis to produce the “fibril reorientation-slippage” hypothesis^34^. The fibril reorientation-slippage hypothesis can explain the decrease in *R* for stage IVb sporangiophores undergoing faster steady elongation growth rates and the increase in *R* during the pressure response. Also it can explain older findings that were previously unexplained^35^. During steady growth it was found that in the lower region of the growth zone (that farthest from the sporangium), rotation rate was measured without measurable elongation rate^35^. But during the light growth response, it was observed that elongation rate was measured in the same lower region that previously showed no elongation rate and in regions below it, i.e. the growth zone was extended and became longer. Furthermore, there was no measureable rotation rate in the extend growth zone region^35^. This interesting behavior can be explained by the fibril reorientation-slippage hypothesis.

### *Global* biophysical equations for expansive growth of walled cells

The sporangiophores of *P. blakesleeanus* (similar to all other fungal, algal, and plant cells) regulate their volumetric growth, cell shape, and sensory responses by controlling the magnitude and direction of the plastic deformation of their cell walls. Volumetric (expansive) growth rates of these walled cells are mediated by wall stress relaxation rates that couple water uptake rate and wall deformation rate^36,37^. Three coupled *global* biophysical equations for the (i) rate of water uptake, (ii) rate of plastic and elastic wall deformation, and (iii) rate of change of turgor pressure have been derived, validated, and established for algal, fungal, and plant cells^38–40^. These global biophysical equations have been extended to plant cells in tissue^41^. Many experiments conducted with the sporangiophores of *P. blakesleeanus* have been used to validate these biophysical equations^42,43^. Relevant to this study is the finding that the turgor pressure did not increase during the light and avoidance growth responses^44^. Furthermore, dimensional analysis has been conducted on these established biophysical equations and dimensionless numbers have been identified that reveal the relative magnitudes of the relevant biophysical processes that occur during expansive growth^45^. These dimensionless numbers can be used to compare the magnitude of biophysical processes in a specific walled cell or compare the magnitude of corresponding processes in different species of walled cells^46,47^. An important dimensionless number, Π_pe_, was identified^48^ that describes the inverse of the dimensionless time constant for stress relaxation of growing cell walls and establishes a quantitative relationship between the steady relative volumetric growth rate, *v*_s_, wall extensibility, *ϕ*, and volumetric elastic modulus, *ε*.

$$ {\Pi }_{{{\text{pe}}}} = \left( {\frac{\varepsilon \phi }{{v_{{\text{s}}} }}} \right) = \left( {\frac{ relative \,\,volumetric\,\,plastic\,\, deformation\,\, rate\,\, of\,\, the\,\, wall}{{relative\,\, volumetric\,\, elastic \,\, deformation\,\, rate\,\, of\,\, the \,\,wall}}} \right) $$

Even though these global biophysical equations have revealed and continue to reveal new insights into expansive growth, they cannot be used to study tropic (bending) responses because they describe the behavior of the whole cell. Bending in a single cylindrical cell requires differential growth rates at opposite sides of the cylindrical cell wall^14^. Thus, modeling the tropic responses requires the development of a *local* biophysical model of the wall that can describe the growth rates in relevant locations of the wall.

### *Local* statistical mathematical model of cell wall extension

The fibril reorientation-slippage hypothesis was used as a conceptual guide for the development of a local statistical mathematical model of the cell wall extension during expansive growth^49^. This model takes a mechanistic view of the “slippage” zone (~ 1000–2500 µm below the base of the sporangium), where two sets of long microfibrils are oriented longitudinally and slip past one another during wall extension. The model describes the elastic and plastic relative displacements between fibrils during elongation growth, stress relaxation, and steps-up in turgor pressure. As the cell wall is viewed as a network of fibrils connected by polymeric tethers, the shearing motions of the fibrils induce tensile stress in the tethers. However, this stress varies among the population of tethers as they are subject to stochastic attachment and detachment events due to biochemical and thermodynamic forces. The molecular mechanisms behind the plastic extension of the wall under stress can be understood as follows. The connecting tethers (a) initially undergo an elastic stretch (the basis of wall elasticity), (b) dissociate from a connecting fibril (resulting in sporadic events of stress relaxation), and (c) re-associate to a different location of the microfibril in a minimum energy configuration. This endows the wall with viscoelastic properties, where viscous flow and elastic deformation are dictated by deformation rate^50^. The model uses a statistical framework to mathematically extrapolate these individual events to a large population of tethers^51–53^. After statistical averaging operations, the model recovers the global biophysical equations for (i) rate of plastic and elastic wall deformation and (ii) rate of change of turgor pressure. The key finding of the statistical model is that global biophysical variables like wall extensibility, *ϕ*, and volumetric elastic modulus, *ε,* could be expressed in terms of molecular properties like tether stiffness *K*, tethers dissociation rate *k*_*d*_ and re-association rate *k*_*a*_. Consequently, the stress relaxation rate of the wall is found to be *k*_*d*_, and the dimensionless number, Π_pe_, can be re-interpreted as

$$ {\Pi }_{{{\text{pe}}}} = \left( {\frac{\varepsilon \phi }{{v_{{\text{s}}} }}} \right) = \left( {\frac{{k_{d} }}{{v_{{\text{s}}} }}} \right) $$

While still a first order approximation that focuses only on fibril-slippage, this model captures the essential molecular features of the cell wall mechanics that can be extended to include fibril-reorientation and helical deformation^54^. Continued development of this local statistical mathematical model requires knowledge of helical growth behavior during bending, i.e. during the phototropic and avoidance responses. Knowledge of how helical growth behaves during these tropic responses will allow a determination of whether the fibril reorientation-slippage hypothesis can explain the helical growth behavior, or if it must be modified in order to provide a conceptual template for the further development of a local statistical mathematical 3-D biophysical model of expansive growth of the cell wall.

### Overview

The first objective of this investigation is to determine whether the elongation rate, rotation rate, and *R* of stage IVb sporangiophores remain constant during the phototropic response and avoidance response. It is found that the elongation and rotation rates increase during the phototropic response, and to a lesser extent during the avoidance response. Similarly, *R* increases during the phototropic response, and to a lesser extent during the avoidance response. Thus the assumption that elongation rates, rotation rates, and *R* are constant during the phototropic response and avoidance response is not supported by these experimental results. The second objective is to determine how the ratio *R* changes with steady elongation rates for stage IVb sporangiophores of the stiff mutants C149 and C216 to learn if they are smaller than *R* of wild type and can offer insight into their diminished phototropic and avoidance responses. The results demonstrate that the “*R* vs. elongation rate” curves for C149 and C216 stiff mutants are similar to that obtained for wild type sporangiophores and not smaller. The ramifications of these results on previous and future research are discussed.

## Results

### Phototropic response

**Figure 3:**
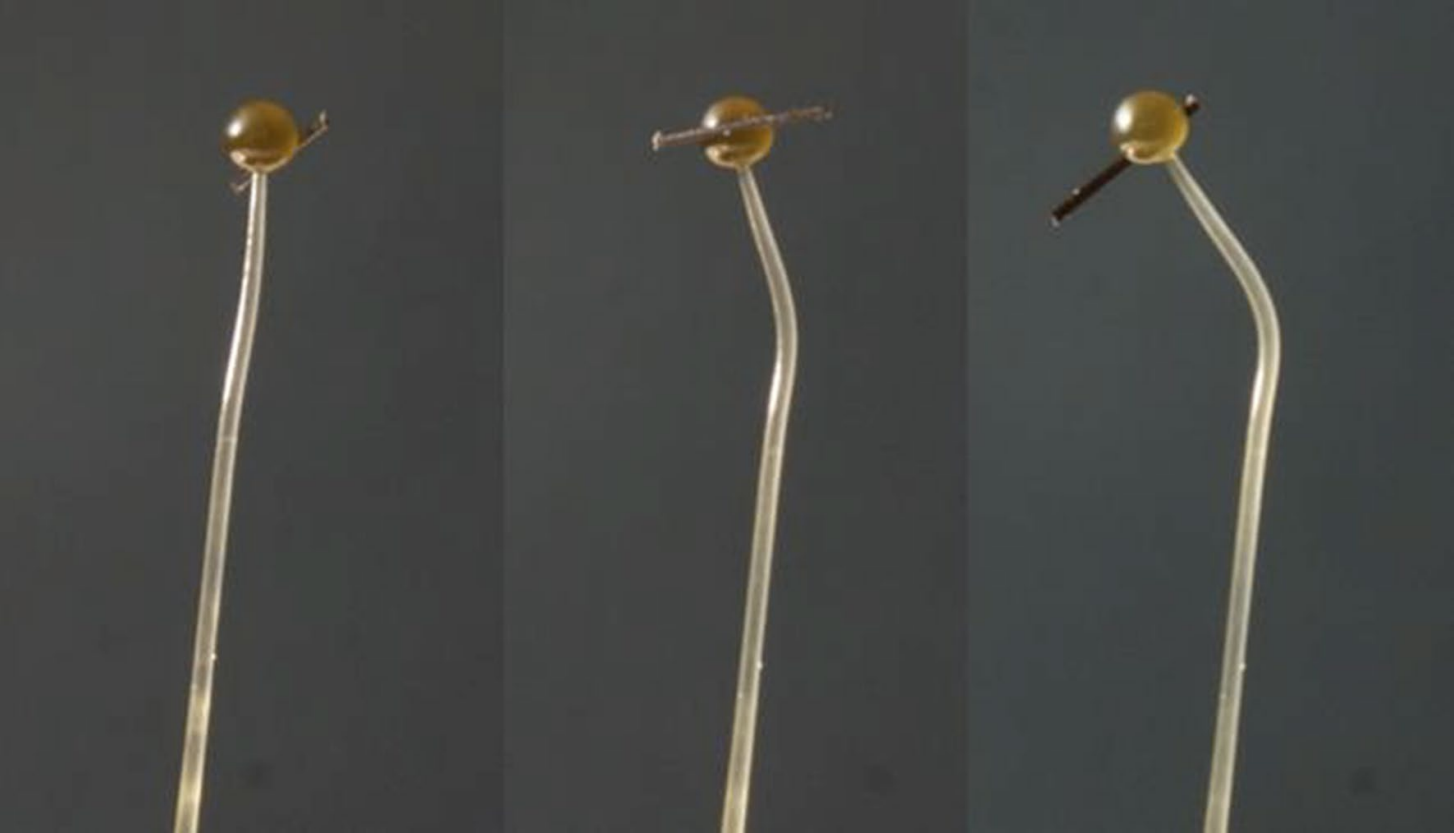
Phototropic response: A stage IVb sporangiophore exposed to a unilateral light stimulus from the left side causing it to bend towards the light source. The three images show the increase in bend angle with time. The light stimulus was initiated after ten minutes of steady growth rate, *t* = 10 min. The first photograph is the sporangiophore at *t* = 10 min. The second and third photographs are taken at *t* = 30 min and *t* = 50 min, respectively. A fiber is attached to the sporangium to determine the rotation rate of the helical growth rate.

Figure 3 show three photographs of the same stage IVb sporangiophore at different times after it was subjected to a unilateral light stimulus from the left side (not shown). A fiber is attached to the sporangium, perpendicular to the longitudinal axis in order to determine the rotation rate of the helical growth rate before and during the phototropic response. It is noted that most of the bending toward the light occurs in the lower region of the growth zone, i.e. that region farthest from the sporangium. This was typical, occurring in eight of ten experiments.

**Figure 4:**
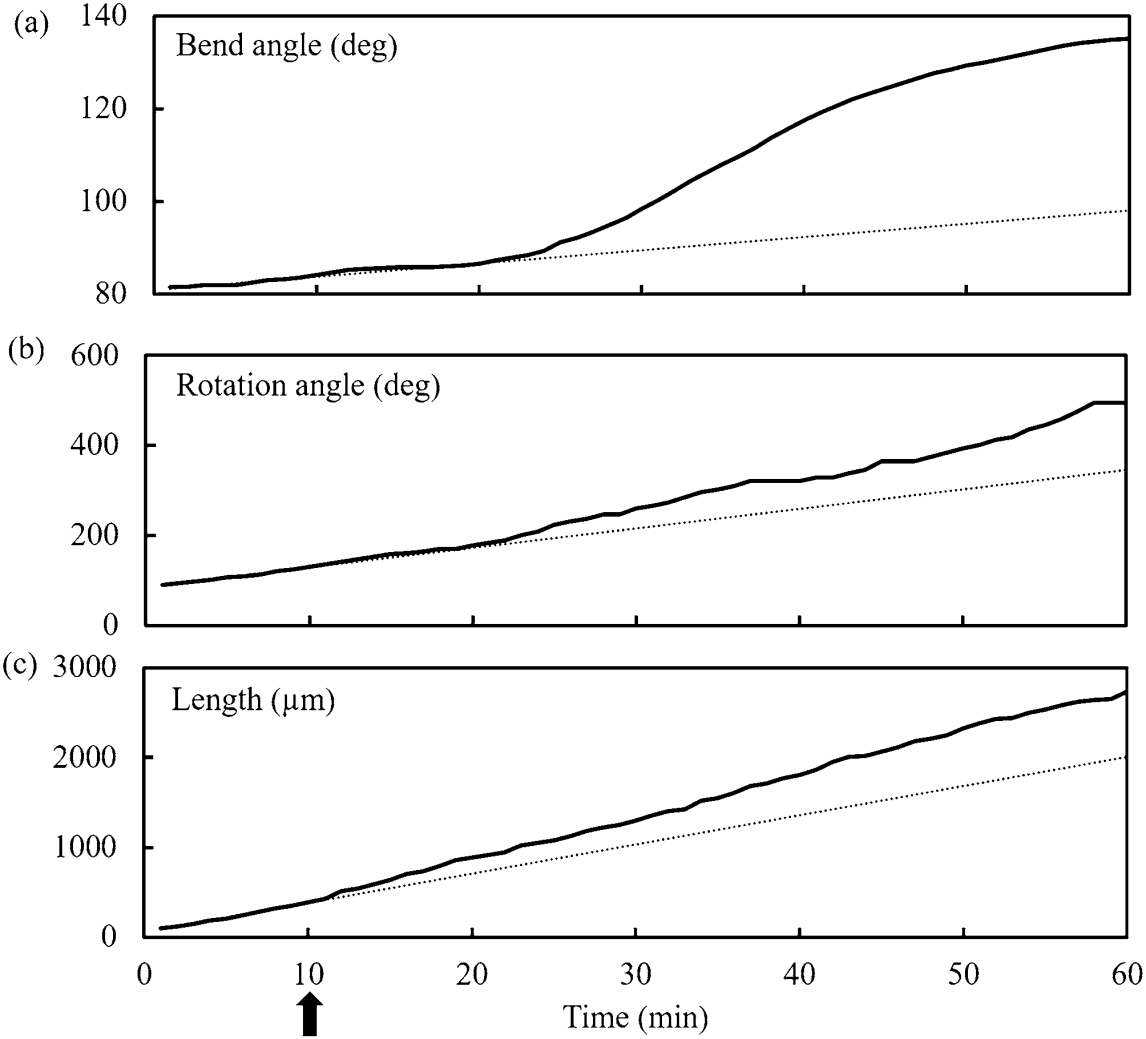
Plots of bend angle (**a**), rotation angle (**b**) and length (**c**) against the same time scale after the start of the experiment for a single stage IVb sporangiophore. The slope of the dashed line in each plot indicates the average bend rate, rotation rate, and elongation rate, respectively, during the first ten minutes of steady growth (before the unilateral light stimulus is given at ten minutes as indicated by the upward arrow). Data obtained from processing photographic images in ImageJ^63^.

Figure 4 shows the results of one experiment where the measured bend angle, rotation angle, and length of the sporangiophore after the start of the experiment are plotted against the same time scale, Fig. 4a–c, respectively. The unilateral light stimulus was given at ten minutes on the time scale and indicated by the upward arrow on the lower time scale. The dashed line is the average slope of the line for the data in the first ten minutes of the experiment, i.e. during steady growth and before the unilateral light stimulus is given. The slopes of the curves are the (a) bend rate, (b) rotation rate, and (c) elongation rate, respectively. In this experiment, it can be seen that the bend rate and rotation rate begin to increase approximately ten minutes after the initiation of the unilateral light stimulus, and the elongation rate begins to increase approximately two minutes afterwards.

**Figure 5:**
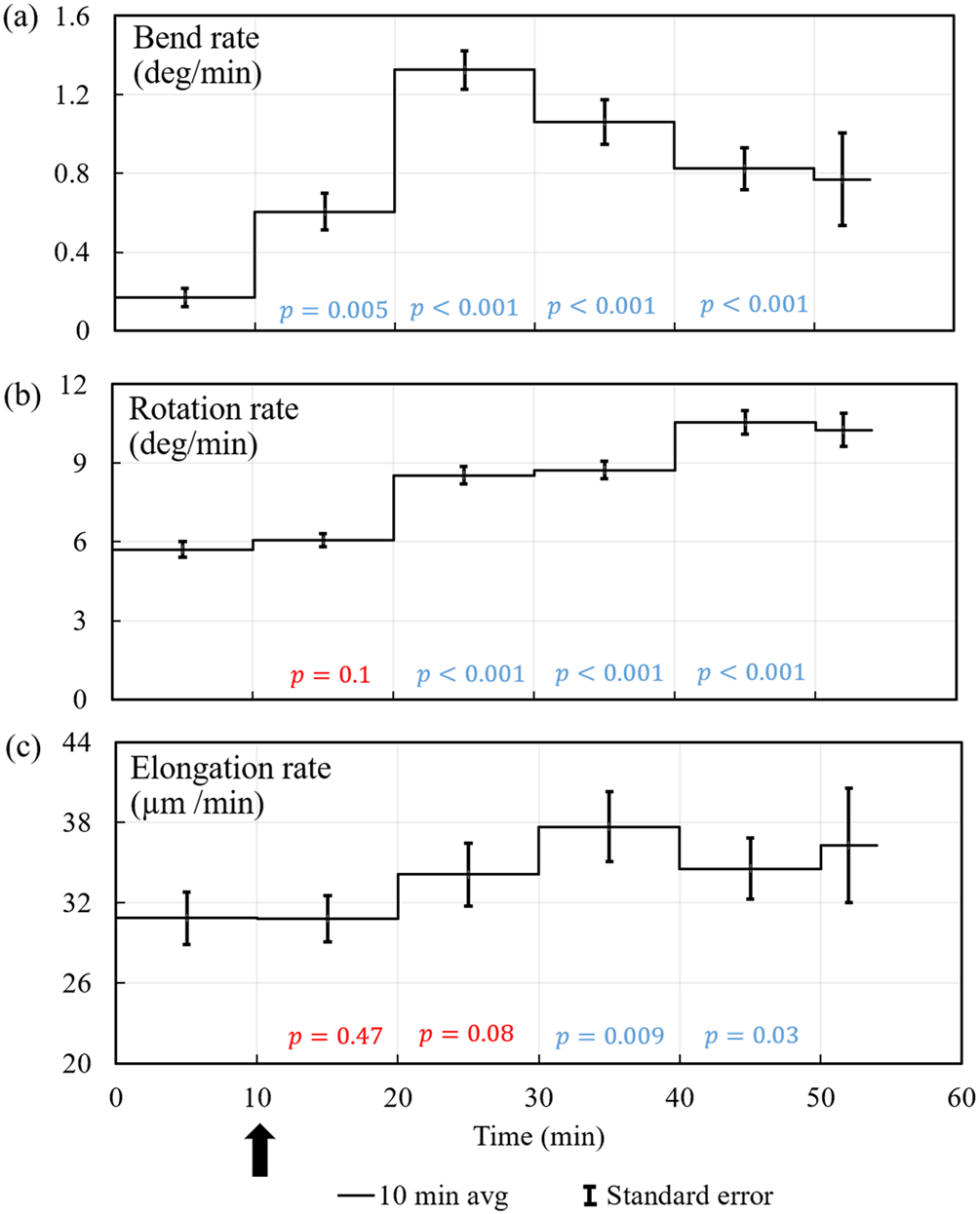
Average bend rate, rotation rate, and elongation rate for each 10 min interval (and for all ten phototropic response experiments) are plotted against the same time scale. The line in each 10 min interval represents the average value for that time interval and the vertical bars indicate the standard error of the mean value. The *p* values on the time scale indicate the degree of confidence that the mean value of the respective time interval is different from the mean of the first 10 min interval, i.e. during steady growth. The *p* values presented on the time scale indicate 95% confidence that the values are different when *p* = 0.05, and smaller values indicate higher confidence that the values are different. The blue *p* values indicate 95% confidence or higher, and the red *p* values indicate less that 95% confidence.

Figure 5 presents the average bend rate, rotation rate, and elongation rate of all ten experiments conducted, Fig. 5a–c respectively. The rates for each ten-minute interval were averaged for all ten experiments and the line in each 10 min interval is the mean value. The vertical bars represent the standard error of the mean value. Student t-tests are conducted to determine whether the mean values for each ten-minute interval after the light stimulus were significantly different than the mean value for the first ten minutes, i.e. during steady growth. The *p* values presented on the time scale indicate 95% confidence that the values are different when *p* = 0.05, and smaller values indicate higher confidence that the values are different (blue type). The red *p* values indicate less than 95% confidence. It can be seen that the rotation rate and elongation rate increase during the phototropic response.

**Figure 6:**
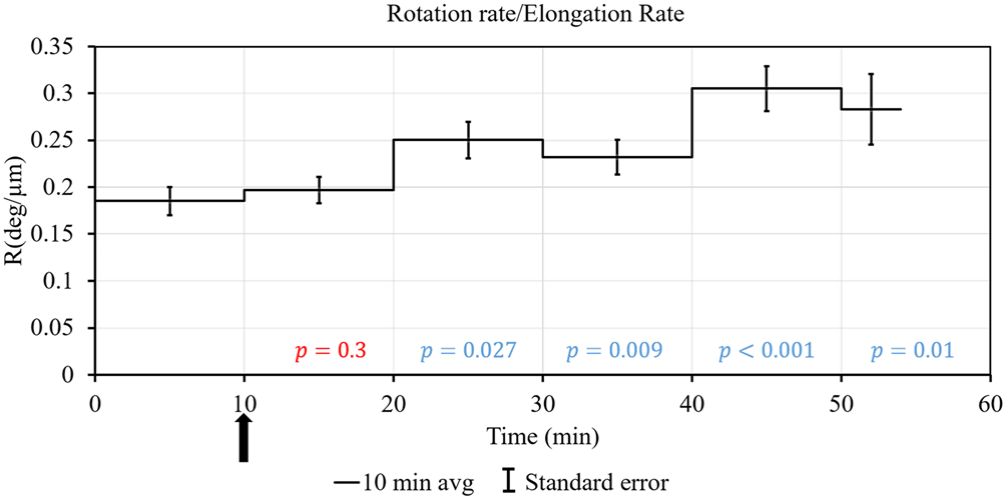
The ratio, *R*, is plotted as a function of time. The horizontal line in each 10 min interval is the mean value, 〈*R*, and the vertical bars represent the standard error of the mean value, *SE*(*R*) as calculated with Eq. (1) and (2), respectively.

The steepness of the helical growth can be determined by the ratio *R* (*R* = rotation growth rate ÷ elongation growth rate). The *R* value was determined for each 10 min interval during the experiments to determine whether the steepness of the helical growth changed during the phototropic response. Figure 6 presents the average *R* values for each 10 min interval for all ten experiments conducted. The “ratio of averages” method was used to calculate *R*. *R* is calculated from their corresponding mean values and standard errors (SE) using the following formulae.

1 $$ \left\langle R \right\rangle = \frac{{\left\langle {RR} \right\rangle }}{{\left\langle {ER} \right\rangle }} $$

2 $$ SE\left( R \right) = R\sqrt {\left( {\frac{{SE\left( {RR} \right)}}{RR}} \right)^{2} + \left( {\frac{{SE\left( {ER} \right)}}{ER}} \right)^{2} } $$

where 〈*R*, 〈*RR*, and 〈*ER* are the mean values for *R*, rotation rate, and elongation rate, respectively.

Figure 6 indicates that the *R* value increases during the phototropic response. Statistics are conducted to determine whether the *R* values during the phototropic response are larger than the *R* value during the first ten-minute interval i.e. steady growth. To determine the statistical significance of the change in *R*, consider the following ratio, *Z*.

3 $$ Z = \frac{{\left\langle R \right\rangle_{t} }}{{\left\langle R \right\rangle_{ref} }} $$

where $\left\langle R \right\rangle_{ref}$ is average value of *R* in the reference time interval (0–10 min) before the stimulus is applied and $\left\langle R \right\rangle_{t}$ represents the average of *R* in a time interval *t* after the stimulus. A mean value of $\left\langle Z \right\rangle \ne 1$, indicates a change in the value of *R* after the application of the stimulus. The standard error in *Z* may be used to calculate confidence intervals for this ratio to determine its statistical significance. Using the same formula for the mean and standard error of a ratio previously defined, the 80% and 95% confidence intervals are calculated for *Z* as $Z \pm 1.25 SE\left( Z \right)$ and $Z \pm 1.96 SE\left( Z \right)$, respectively. Table 1 summarize these confidence intervals for the phototropic response.

**Table 1:** The 80% and 95% confidence intervals that *R* increases during the phototropic response are presented.

Time range (min)	Average Z	80% confidence interval	95% confidence interval
10–20	1.06	0.92–1.21	0.83–1.29
20–30	1.35	1.16–1.54	1.16–1.54
30–40	1.25	1.07–1.43	1.07–1.43
40–50	1.65	1.42–1.88	1.28–2.02
50–60	1.53	1.83–1.23	1.06–2.00

It is noted that *Z* is greater than unity (*Z* > 1) for the time intervals greater than 20 min for both 80% and 95% confidence intervals. Thus the results indicate 95% confidence that *R* has increased during the time interval of 20–60 min of the phototropic response.

### Avoidance response

**Figure 7:**
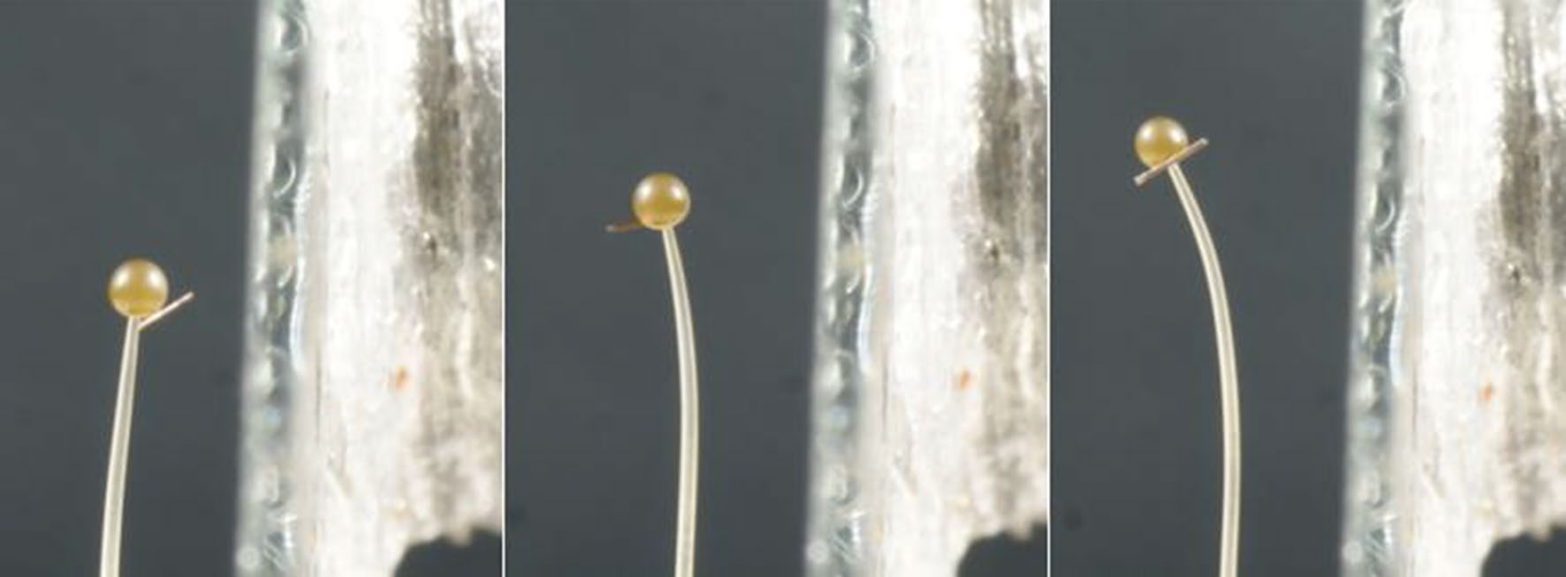
Avoidance response: A stage IVb sporangiophore exposed to a barrier stimulus from the right side (a microscope slide cover slip) causing it to bend away from the barrier. The three images show the increase in bend angle with time. The barrier stimulus was initiated after ten minutes of steady growth rate, *t* = 10 min. The first photograph is the sporangiophore at *t* = 10 min. The second and third photographs are taken at *t* = 30 min and *t* = 50 min, respectively. A fiber is attached to the sporangium to determine the rotation rate of the helical growth rate.

Figure 7 show three photographs of the same stage IVb sporangiophore at different times after it was subjected to a barrier stimulus from the right side (a microscope slide cover slip is advanced to within approximately 0.7 mm of the sporangiophore stalk without touching it). A fiber is attached to the sporangium, perpendicular to the longitudinal axis in order to determine the rotation rate of the helical growth rate before and during the avoidance response. It is shown that the bending occurs gradually throughout the growth zone. This was typical, occurring in all ten experiments.

**Figure 8:**
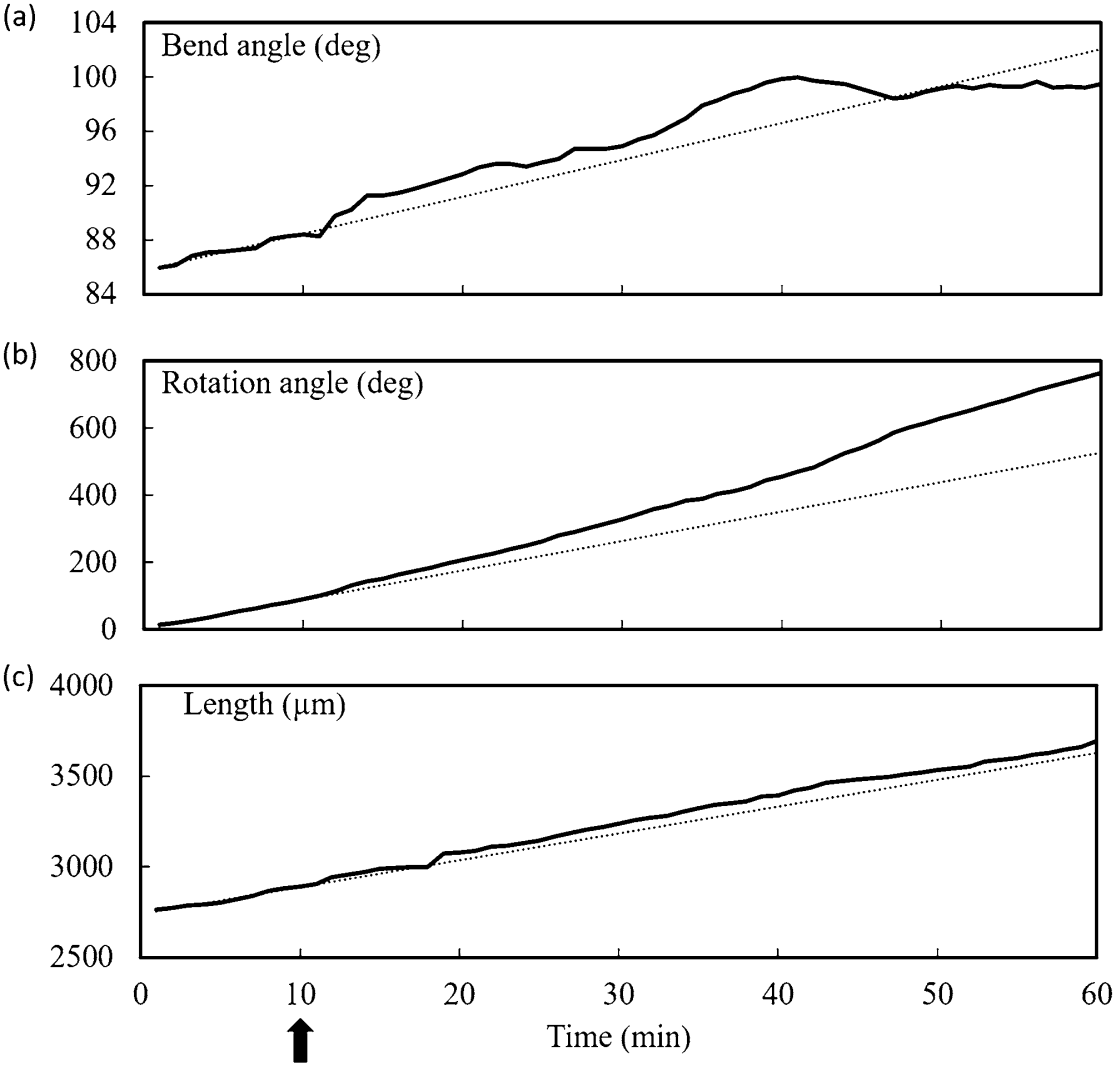
Plots of bend angle (**a**), rotation angle (**b**) and length (**c**) against the same time scale after the start of the experiment for a single stage IVb sporangiophore. The slope of the dashed line in each plot indicates the average bend rate, rotation rate, and elongation rate, respectively, during the first ten minutes of steady growth (before the unilateral light stimulus is given at ten minutes as indicated by the upward arrow). Data obtained from processing photographic images in ImageJ^63^.

Figure 8 shows the results of one experiment where the measured bend angle, rotation angle, and length of the sporangiophore after the start of the experiment are plotted against the same time scale, Fig. 8a–c, respectively. The barrier stimulus was given at ten minutes on the time scale and indicated by the upward arrow on the lower time scale. The dashed line is the average slope of the line for the data in the first ten minutes of the experiment, i.e. during steady growth and before the barrier stimulus is given. The slopes of the curves are the (a) bend rate, (b) rotation rate, and (c) elongation rate, respectively. In this experiment, it can be seen that the bend rate, rotation rate, and elongation rate begin to increase approximately two to three minutes after the initiation of the barrier stimulus.

**Figure 9:**
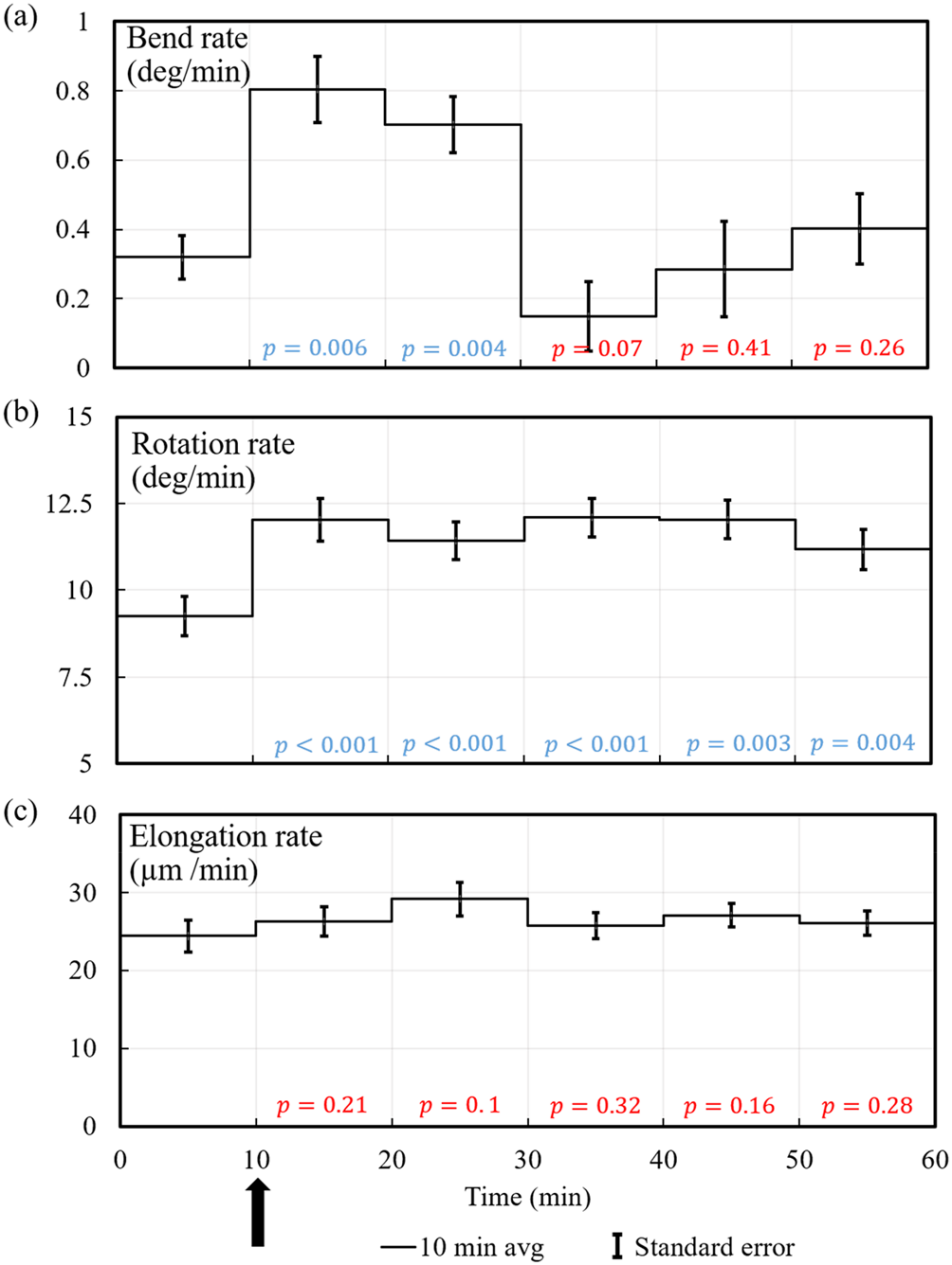
Average bend rate, rotation rate, and elongation rate for each 10 min interval of the experiment are plotted against the same time scale for all ten avoidance response experiments. The line in each 10 min interval represents the average value for that time interval and the vertical bars indicate the standard error of the mean value. The *p* values on the time scale indicate the degree of confidence that the mean value of the respective time interval is different from the mean of the first 10 min interval, i.e. during steady growth. The *p* values presented on the time scale indicate 95% confidence that the values are different when *p* = 0.05, and smaller values indicate higher confidence that the values are different. The blue *p* values indicate 95% confidence or higher, and the red *p* values indicate less that 95% confidence.

Figure 9 presents the average bend rate, rotation rate, and elongation rate of all ten experiments conducted, Fig. 9a–c, respectively. The rates for each ten-minute interval were averaged for all ten experiments and the line in each 10 min interval is the mean value. The vertical bars represent the standard error of the mean value. Student t-tests are conducted to determine whether the mean values for each ten-minute interval after the barrier stimulus were significantly different than the mean value for the first ten minutes, i.e. during steady growth. The *p* values presented on the time scale indicate 95% confidence that the values are different when *p* = 0.05, and smaller values indicate higher confidence that the values are different. The blue *p* values indicate 95% confidence or higher, and the red *p* values indicate less that 95% confidence. It is seen that the rotation rate significantly increases during the avoidance response, but the elongation rate does not.

**Figure 10:**
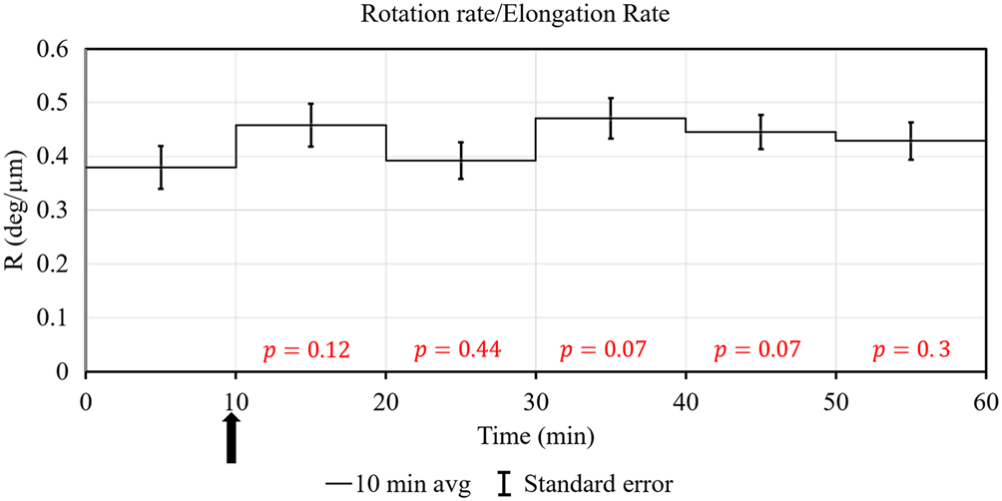
The ratio, *R*, is plotted as a function of time. The horizontal line in each 10 min interval is the mean value, 〈*R*, and the vertical bars represent the standard error of the mean value, *SE*(*R*) as calculated with Eqs. (1) and (2), respectively.

Figure 10 presents the average *R* values for each 10 min interval for all ten experiments conducted. As before, the “ratio of averages” method was used to calculate *R* from their corresponding mean values and standard errors (SE) using Eqs. (1) and (2).

The same statistical method used for the phototropic response was used to determine whether the *R* values during the avoidance response are larger that the *R* value during the first ten minute interval i.e. steady growth. Equation (3) was used to calculate *Z* to determine the 80% and 95% confidence intervals, $Z \pm 1.25 SE\left( Z \right)$ and $Z \pm 1.96 SE\left( Z \right)$, respectively. Table 2 summarize these confidence intervals for the avoidance response.

**Table 2:** The 80% and 95% confidence intervals that *R* increases during the avoidance response are presented.

Time range (min)	Average Z	80% Confidence interval	95% Confidence interval
10–20	1.20	1.00–1.41	0.88–1.53
20–30	1.03	0.85–1.21	0.76–1.31
30–40	1.24	1.03–1.44	0.92–1.56
40–50	1.17	0.99–1.36	0.88–1.46
50–60	1.13	0.94–1.32	0.84–1.42

It is noted that *Z* is less than unity (*Z* < 1) for the all time intervals in the 95% confidence interval. *Z* is greater than unity in the 80% confidence interval for time intervals 10–20 min and 30–40 min. This result indicates only 80% confidence that *R* has increased during the time intervals of 10–20 and 30–40 min of the avoidance response.

### *R* versus elongation rate for “stiff mutants”

**Figure 11:**
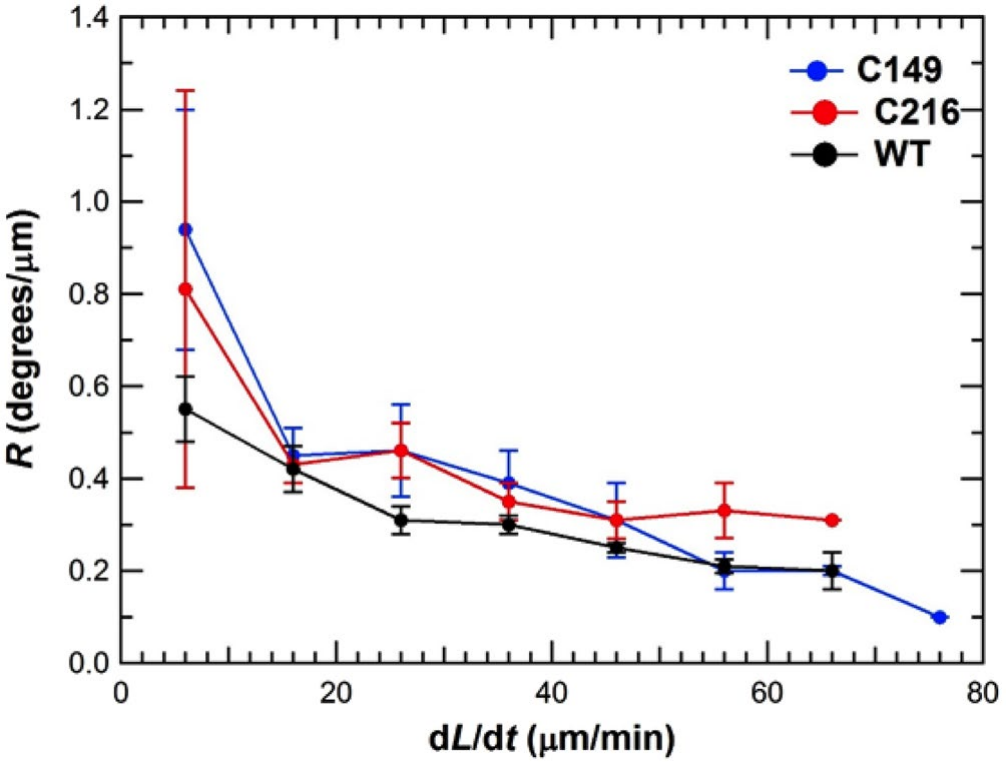
Average *R* values versus elongation rates for stage IVb sporangiophores of wild type (black curve) and stiff mutant strains, C149 (blue curve) and C216 (red curve). Each point represents the mean value of *R* for sporangiophores with steady elongation rates in ten minute intervals: 1–10.9 μm/min ……. 61–70.9 μm/min. The vertical bars are the standard errors (SE) of the mean value. There were 59 sporangiophores tested for stiff mutants and 171 sporangiophores for wild type. The curve “*R* versus elongation rate” for wild type stage IVb sporangiophores (black curve) was obtained from Ortega et al., 2003 with permission. The curves “*R* versus elongation rate” for stiff mutants stage IVb sporangiophores (blue and red curves) were obtained from Munoz^55^ with permission.

*R*_ave_ was determined for stiff mutant stage IVb sporangiophores (C149 and C216) during steady elongation growth using the same method and analyses that were previously used for wild type sporangiophores^34^. The elongation and rotation rates were measured concurrently for mutant stage IVb sporangiophores during steady growth. The natural variation of steady elongation growth rate ranged between approximately 5 μm/min and 76 μm/min. The ratio, *R*, was calculated for each sporangiophore and averaged with values of *R* obtained from other sporangiophores growing within a specified range of elongation rates. These averaged values, *R*_ave_, are plotted together with those obtained from wild type stage IVb sporangiophores as a function of elongation rate in Fig. 11. The three curves appear similar both visually and statistically. However, student t-tests to determine whether the data points for each time interval were statistically different or the same could not be conducted because the data for the wild type sporangiophores was unavailable. However, it is shown that the curves for the mutant sporangiophores, C149 and C216, are generally higher (have larger *R* values) than that for the wild type sporangiophores^55,56^.

## Discussion

### Phototropic response

Figure 3 shows a stage IVb sporangiophore growing and bending toward a light source (not shown) that is located on the left side. In this experiment all the bending occurred in the lower region of the growth zone. This sharp and localized bending in the lower region of the growth zone occurred in eight out of ten experiments. In the two remaining experiments the bending was gradual as shown in Fig. 7. It is not known why the different type of bending occurs. Figure 4 show the bend angle, rotation angle, and elongation that occurs after the initiation of the experiment for a single stage IVb sporangiophore. It can be seen in Fig. 4a that the bend rate (slope of the line) begins to increase approximately ten minutes after the unilateral light stimulus was given, but this was not typical. Usually the latency for the response was about four minutes. This example was included to show the variety of results obtained. Figure 4b shows that the rotation rate also begins to increase at approximately ten minutes. Figure 4c shows that the elongation rate begins to increase approximately two minutes after the initiation of the unilateral light stimulus.

The average bend rate, rotation rate, and elongation rate of all ten experiments conducted are presented in Fig. 5. It can be seen that the bend rate increases during the first ten-minute interval after the light stimulus with a maximum occurring during the second ten-minute interval. The average rotation rate also increases during and throughout the phototropic response. The average elongation rate increased during the third and fourth ten-minute intervals. The results demonstrate that both the rotation rate and elongation rate increase during the phototropic responses.

Figure 6 presents the average *R* values for each ten-minute interval for all ten experiments conducted. Table 1 indicates that the average *R* values are significantly larger for the second, third, and fourth ten-minute intervals. It is concluded that the average *R* value increases during the phototropic response. The results indicate that the steepness of the helical growth rate becomes flatter during the phototropic response.

Castle^14^ assumed constant elongation rates during the phototropic response. It was postulated that bending was produced by differential growth between the proximal and distal sides of the sporangiophore stalk. It was assumed that the proximal wall decreases in elongation rate and the distal wall increases in elongation rate, so that the cell’s overall elongation rate was constant. This assumption is not supported by the results presented in Fig. 5. Here it is learned that the overall elongation rate increases during the phototropic response. It appears that differential growth produced by an increase in elongation rate of the distal side (but not a decrease in elongation rate of the proximal side) will produce the bending and increase in overall elongation rate that are shown in Fig. 5. It may be that the molecular stimulant diffuses to the proximal side, but is concentrated on the distal side, and produces slightly larger elongation rate in addition to the bending. Otherwise, the main conclusions by Castle^14^ remain the same. Similarly, the finding that both elongation and rotation rates increase during the phototropic response do not appear to alter the main conclusions of Dennison and Foster^15^, that the rotation converts a spatial stimulus into a temporal stimulus that prevents adaptation to the unilateral light stimulus.

### Avoidance response

Figure 7 shows a stage IVb sporangiophore growing and bending away from a solid barrier (microscope slide) that is located on the right side. Here, the microscope cover slip is positioned parallel to the growth zone of the sporangiophore and parallel to the light from the sides, so that it does not interfere with the light. The barrier is positioned to be within 1 mm of the surface but not in contact. In this experiment the bending was gradual and appears to occur over the length of the growth zone. This was typical of the ten experiments conducted for the avoidance response. Figure 8 show the bend angle, rotation angle, and elongation that occurs after the initiation of the experiment for a single stage IVb sporangiophore. It can be seen in Fig. 8 that the bend rate, rotation rate, and elongation rate (i.e. the slope of the lines) begins to increase approximately 1–2 min after the avoidance stimulus was given. This was typical.

The average bend rate, rotation rate, and elongation rate of all ten experiments conducted are presented in Fig. 9. It can be seen that the bend rate increases during the first and second ten-minute interval after the avoidance stimulus was given, with a maximum occurring during the first ten-minute interval. In this experimental arrangement, the sporangiophore grows out of range of the barrier stimulus. The avoidance response would continue if the barrier were continual moved to maintain a close distance (~ 0.7 mm) to the sporangiophore’s growth zone. Figure 9 shows that the rotation rate increases during the avoidance response and afterwards, i.e. during the second through fifth ten-minute intervals. The elongation rate appears to increase slightly during the second ten-minute interval of the avoidance response (90% confidence interval). It is concluded that the rotation rate increases during the avoidance response and there may be a small increase in elongation rate during the avoidance response.

Figure 10 presents the average *R* values for each ten-minute interval for all ten experiments conducted. Table 2 indicates that the average *R* values may be larger for the first and third ten-minute intervals (80% confidence interval). It is concluded that the *R* value may slightly increases during the avoidance response.

A comparison of the curves in Figs. 5a and 9a demonstrate that the strength, or bending rate, of the phototropic response is larger than that of the avoidance response (1.3 deg/min compared to 0.8 deg/min). This difference may account for the smaller increase in elongation rate and *R* value during the avoidance response. A stronger response can be obtained by putting the barrier closer to the sporangiophore^17^, but this was not possible in these experiments because it would constrain the rotation of the fiber on the sporangium. The observation that the sporangiophores exhibit sharp curvature in the lower region of the growth zone during the phototropic response, but not during the avoidance response, may indicate that there may be underlining differences between these tropic responses.

### *R* versus elongation rate for stiff mutant sporangiophores; C149 and C216

A distinguishing feature between “growth” responses and “tropic” responses is that growth responses are transient but tropic responses can continue indefinitely, as long as an effective stimulus can be maintained. In the phototropic response, it has been hypothesized that the photoreceptors rotate with the cell wall, and this rotation prevents the photoreceptors from adapting to the light stimulus^15^. This wall rotation argument is extended to the avoidance response^16^ and may be extended to the gravitropic response that can also continue indefinitely. A question that arises is whether the diminished tropic responses of the stiff mutants are the result of a diminished rotation rate of the wall in the growth zone. Little is known about the helical growth of these stiff mutants.

The average elongation growth rates of C216 and C149 stage IVb sporangiophores are similar to those of wild type^56^. However, the length of the growth zones for the C216 and C149 sporangiophores are shorter than wild type sporangiophores. So it is speculated that the shorter growth zones of the stiff mutant sporangiophores may reduce the overall rotation rate that could contribute to the diminished tropic responses. Therefore, *R* was measured as a function of steady elongation rate for both C216 and C149 stage IVb sporangiophores.

The curves of *R* as a function of elongation rate for C149, C216, and wild type (WT) stage IVb sporangiophores are presented Fig. 11. The results indicate that for C149 and C216 sporangiophores, *R* decreases as the elongation rate increases, similar to wild type sporangiophores. But the results clearly demonstrate that the *R* values for C149 and C216 stiff mutant stage IVb sporangiophores not smaller than those of wild type^55^. Therefore, it is concluded that the mutation that produces the diminished phototropic and avoidance responses are not mutations in the helical growth of their sporangiophores. It is still possible that the shorter growth zones of these stiff mutant sporangiophores may contribute to the diminished tropic responses^56^.

### Helical growth

The fibril reorientation hypothesis was proposed to explain the origin of helical growth in the elongating cell wall in the growth zone^30,32,35^. It is proposed that microfibrils are deposited somewhat transverse to the longitudinal axis on the inner wall surface of the growth zone with a concentration in the upper region of the growth zone, that nearest the sporangium. The upper region of the growth zone is shown to be the most extensible and exhibits the largest amount of plastic deformation^57^. It is thought that the transverse orientation of microfibrils allows for more resistance to wall deformation in the radial direction compared to the longitudinal direction. It is postulated that because of longitudinal plastic wall deformation and deposition of new materials to the inner wall during elongation growth, the microfibrils reorient towards the longitudinal direction and concurrently migrate to lower regions of the growth zone. Now if the fibril reorientation occurs in a preferred direction because of some asymmetry in the molecular bonding or because of its initial orientation, then the displacement vector of the reorienting microfibrils will produce transverse displacement on the elongating (stretching) wall (Fig. 2a). In a cylinder, the transverse displacement is converted to rotation around the longitudinal axis. The concurrent rotation rate and elongation rate produce the helical growth rate that is observed. This hypothesis predicts that the orientation of the microfibrils will be somewhat transverse oriented in the upper region of the growth zone nearest the sporangium and mostly longitudinal oriented in the lower region farthest from the sporangium. This prediction is supported by experimental observation^25,26,58^. Because of the different orientation of the microfibrils along the length of the growth zone, the amount of transverse displacement (rotation) per longitudinal displacement produced by fibril reorientation changes along the length of the growth zone. The amount of rotation per longitudinal displacement increases as the microfibrils become more longitudinally oriented. Importantly, the fibril reorientation hypothesis predicts that the ratio of the rotation rate and elongation rate, *R*, increases in the lower region of the growth zone. This prediction is consistent with experimental results indicating that there is measurable rotation without measurable elongation in lower region of the growth zone during steady elongation growth^30,35^.

*Pil*-mutant stage IVb sporangiophores exhibit increased radial expansion during elongation growth and form a bulge in the growth zone of the stage IVb sporangiophore^33^. Interestingly, the direction of rotation of the wall switches from clockwise to counter-clockwise during the increase in radial expansion. It is postulated that the mutation is that the microfibrils are deposited somewhat longitudinally, instead of the transverse direction, on the inner wall surface of the growth zone. The longitudinally oriented microfibrils will permit more wall deformation and strain in circumference, thus initiating radial expansion. It is thought that the microfibrils will begin to reorient toward the transverse direction because of the increased wall deformation in the circumferential direction. If the direction of fibril reorientation is the same as before (during elongation growth) but simply reversed, then the transverse wall displacement caused by the reorienting microfibrils will be in the opposite direction and the wall rotation will be counterclockwise. The fibril reorientation hypothesis predicts that the reversal from clockwise to counterclockwise will occur when the rate of wall deformation in the circumferential direction and longitudinal direction are equal. Analyses of the experimental results confirm this prediction^33^.

The findings that *R* is a function of elongation rate^34^ (Fig. 11, black curve) and *R* increased during the pressure response were the incentive to modify the fibril reorientation hypothesis and postulate the fibril reorientation-slippage hypothesis. Prior research demonstrates that sporangiophores with larger elongation growth rates also have longer growth zones^59,60^, and an equation was proposed that relates the elongation rate with the length of the stage IVb growth zone^60^. It is envisioned that the reorienting microfibrils will reorient completely toward the longitudinal axis in the lower regions of the longer growth zone, after which longitudinal wall deformation can only occur by microfibrils sliding past each other (fibril slippage). It is postulated that there exists a fibril reorientation subzone and fibril slippage subzone in longer, faster elongating growth zones and the fibril slippage zone does not produce transverse wall displacement or rotation. The fibril reorientation-slippage hypothesis predicts that during fast steady growth, longer growth zones will have a fibril slippage subzone. Furthermore, longer growth zone with both fibril reorientation and fibril slippage subzones will produces less average rotation compared to a shorter growth zone with only a fibril reorientation subzone. In other word, it predicts that the ratio of rotation rate and elongation rate, *R*, will be smaller for faster growing sporangiophores^34^ (Fig. 11). Previously, it was shown that the pressure response (a transient decrease in elongation rate) was accompanied by a decrease in the length of the growth zone^10^. The fibril reorientation-slippage hypothesis predicts that a shorter growth zone will produce more total rotation and thus a larger *R*. Both of these predictions are consistent with the experimental finding^34^. In addition, the fibril reorientation-slippage mechanism can explain why “measureable rotation rate without measureable elongation rate” is observed during steady growth in the lowest region of the growth zone (that most distant from the sporangium) and why “measureable elongation rate without measureable rotation rate” is observed during the light growth response (transient increase in elongation growth) in the lowest region of a longer growth zone^35^.

In this study, the rotation rate, elongation rate, and *R* are measured during two tropic responses, the phototropic and avoidance responses. It is found that the rotation rate increases during both tropic responses. The elongation rate increases during the phototropic response and appears to increase slightly during the avoidance response. Similarly *R* increases during the phototropic response and appears to increase slightly during the weaker avoidance response. Here we ask whether the fibril reorientation-slippage hypothesis can explain the behavior of the rotation rate, elongation rate, and *R* that is observed during the phototropic and avoidance responses. It is thought that the increase in elongation growth rate during the phototropic response is produced by an increase in wall loosening (mediated by a wall-loosening proteins and/or enzymes) that increases the plastic wall deformation. If it were assumed that the microfibrils are added to the inner wall at the same rate and location as in steady growth, then the fibril reorientation-slippage hypothesis would predict a *decrease* in the magnitude of rotation rate and *R* during the phototropic response. This is expected because the increase in elongation rate will be accompanied by an increase in growth zone length and most of the added length will be fibril-slippage subzone that does not produce rotation. On the other hand, if it is assumed that microfibrils are added to the inner wall at a higher rate and to larger (lower) areas of the growth zone during the phototropic response, then the fibril reorientation-slippage model will predict an *increase* in rotation rate and *R*, as shown in Fig. 5 and Table 1. Prior research has demonstrated that chitin synthesis is stimulated by an increase in light intensity^61^. This would increase the population of reorienting microfibrils in the lower region of the longer growth zone, thus converting the “fibril slippage” subzone to a “fibril reorientation” subzone. The reorienting fibrils will continue to produce rotation in the lower region and increase the overall rotation rate and *R* during the phototropic response.

The fibril reorientation-slippage model can explain the experimental results obtained in this study if it is postulated that the phototropic response is initiated by producing additional wall loosening on the distal side of the cylindrical wall of the sporangiophore. Then the distal wall would experience more loosening than the proximal wall, producing bending toward the unilateral light and increasing the overall elongation rate. When the sporangiophore approaches a barrier, the proximal wall will experience additional wall loosening, producing bending away from the barrier and increasing the overall elongation rate. In both cases, it is postulated that the production rate of microfibrils in a transverse direction is increased and extended to *lower regions* of a slightly longer growth zone (the larger elongation rates are accompanied by a longer growth zones^60^). It is envisioned that the increase in population of reorienting microfibrils in lower regions of the growth zone will effectively convert some of the fibril-slippage subzone into a fibril-reorientation zone that produces rotation. Recent evidence indicates that the addition of polysaccharide chains to synthesized microfibrils may occur below the growth zone^62^. The introduction of a population of reorienting microfibrils in the lower regions of the growth zone will increase the rotation rate and the magnitude of *R* as shown in Fig. 5b and Table 1, and in Fig. 9b and Table 2.

**Figure 12:**
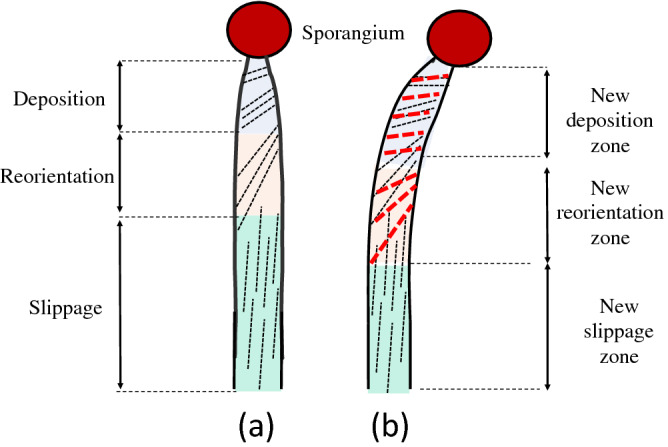
The postulated fibril reorientation-slippage model undergoing large elongation rates during (**a**) steady growth and (b) the phototropic response. The black dashed lines in (**a**) represent the hypothesized direction of the microfibrils at different locations of the growth zone in a fast growing sporangiophore. It is envisioned that the microfibrils are deposited in a somewhat transverse direction (deposition zone) and begin to reorient toward the longitudinal direction because of the longitudinal deformation that occurs during elongation growth (reorientation zone). Rotation is produced in the reorientation zone. In a fast growing sporangiophore it is envisioned that the fibrils reorient to the longitudinal direction in the lower section of the growth zone and can only slide by each other without producing rotation (slippage zone). During the phototropic response (**b**), it is hypothesized that there is an increase in the rate of deposition of microfibrils (red dashed lines) and the deposition zone is increased in size (length). The increase in the deposition zone produces an increase in the length of the reorientation zone that extends into the fibril slippage zone. This in turn produces an increase in rotation rate during the phototropic response.

Figure 12 is a schematic illustration of the postulated fibril reorientation-slippage model undergoing large elongation rates during (a) steady growth and (b) the phototropic response.

In the near future, we plan to test these predictions using a *local* model of the cell wall that is being developed^49,54^ to explore how fibril reorientation, fibril slippage, and the addition of wall material produce helical growth, and the changes in helical growth that occurs during sensory responses.

### *Global* biophysical equations for expansive growth of walled cells

The global biophysical equations^38–41^ implicitly assume that mass is added to the cell wall during expansive growth to prevent thinning and eventual rupture of the wall. In order to maintain a constant wall thickness during steady growth rate, the rate at which mass is added to the wall is considered to be constant, i.e. (d*m*_w_/d*t*)_ss_ = constant. It is not known how the wall mass changes during sensory responses or which species of biomolecules and polymers are added. Here we present evidence that there is an increase in the rate of production of chitin microfibrils during the phototropic and avoidance responses. So in general, Eq. (4) can be added to the three global biophysical equations to explicitly describe the addition of wall mass during sensory responses (SR).

4 $$ \frac{{{\text{d}}m_{{\text{w}}} }}{{{\text{d}}t}} = \left( {\frac{{{\text{d}}m_{{\text{w}}} }}{{{\text{d}}t}}} \right)_{{{\text{SS}}}} + \left( {\frac{{{\text{d}}m_{{\text{w}}} }}{{{\text{d}}t}}} \right)_{{{\text{SR}}}} $$

Then for the phototropic response (PR) we can write a more explicit equation, Eq. (5), when there are *i*-species of biomolecules and polymers added to the wall.

5 $$ \frac{{{\text{d}}m_{{\text{w}}} }}{{{\text{d}}t}} = \left( {\frac{{{\text{d}}m_{{\text{w}}} }}{{{\text{d}}t}}} \right)_{{{\text{SS}}}} + \left( {\frac{{{\text{d}}m_{{\text{chitin fibils}}} }}{{{\text{d}}t}}} \right)_{{{\text{PR}}}} + \mathop \sum \limits_{n = 2}^{n = i} \left( {\frac{{{\text{d}}m_{i} }}{{{\text{d}}t}}} \right)_{{{\text{PR}}}} $$

Similarly for the avoidance response (AR) we can write Eq. (3), when there are *j*-species of biomolecules and polymers added to the wall.

6 $$ \frac{{{\text{d}}m_{{\text{w}}} }}{{{\text{d}}t}} = \left( {\frac{{{\text{d}}m_{{\text{w}}} }}{{{\text{d}}t}}} \right)_{{{\text{SS}}}} + \left( {\frac{{{\text{d}}m_{{\text{chitin fibils}}} }}{{{\text{d}}t}}} \right)_{{{\text{AR}}}} + \mathop \sum \limits_{n = 2}^{n = j} \left( {\frac{{{\text{d}}m_{j} }}{{{\text{d}}t}}} \right)_{{{\text{AR}}}} $$

The number of species of biomolecules and polymers added to the cell wall during the phototropic response (n = *i*) and during the avoidance response (n = *j*) are unknown at this time. However, as they become known they can be added to Eqs. 2 and 3, making the rate of change of cell wall mass more explicit.

### *Local* statistical mathematical model of cell wall extension

A better understanding of how walled cells regulate the magnitude and direction of the wall deformation to obtain different shapes or produce sensory responses requires a *local* biophysical model of wall deformation. Other growth behavior such as helical growth and reversal of rotation during stage IV also require a local model of wall deformation. Statistical mechanics can be a powerful tool to translate local biophysical features and mechanisms involving the wall components like fibril stiffness and bond dynamics to global changes in deformation and growth. Even when restricted to pure fibril slippage^49^, the statistical model has provided important insights on the biophysical relationship between local bond dynamics and stress relaxation and growth rate of the wall during a pressure response. Consequently, it is found that dimensionless numbers like Π_pe_, which is the ratio of plastic to elastic rates of wall deformation, is directly related the rate of bond dissociation. A local statistical model can, therefore, provide a quantitative means to test the fibril-reorientation-slippage hypothesis with the distinct advantage of directly incorporating molecular mechanisms in the wall structure. Such a model will not only describe the varying fibril orientation across the growth zone, but also incorporate changes in mass deposition rate and regulation of bond dynamics at different stages of growth and particularly during sensory responses.

While the general mathematical framework for a local statistical model has been developed^54^, its true potential cannot be unlocked without the guidance of experimental observations and the right hypotheses for the underlying molecular mechanisms. The results reported on the change of *R* during the phototropic and avoidance responses provide crucial data that will be used to validate the predictions of the local model. For example, the leading hypothesis of increased rate of fibril deposition on the inner wall on the lower part of the growth zone during these sensory responses can be tested by comparing the corresponding predictions of *R* emerging from the local model. Furthermore, the relationship between the length of the growth zone and the resulting sensory response can be investigated further using the observations of stiff mutants and wild type sporangiophores. Establishing a feedback loop between experimental observations and model predictions can thus lead to quantitative confirmation of existing hypotheses and discovery of new ones about the molecular features of the cell wall.

## Materials and methods

### Phototropic and avoidance response experiments

#### Biological material

Hypha were purchased from Carolina Biological supply in the form of *Phycomyces blakesleeanus* (-), Living, Tube Culture, from which a small amount was cultivated on a petri dish of prepared 4% potato dextrose agar. Spores were obtained from the sporangiophores and stored in distilled water and saved. The growth medium was prepared from potato dextrose agar manufactured by Alpha Biosciences. Vegetative spores were inoculated in the culture medium and grown in small glass vials (capacity: 1 g) in a box at a temperature of 23 °C and high humidity. After the appearance of sporangiophores, they were plucked (tall sporangiophore were pulled from the mycelium by hand) every evening so that a fresh crop of sporangiophores would be available the next day. Stage IVb sporangiophores, 2–5 cm in length, from the third to fifth crop was used for experimentation.

#### Phototropic response experiments

To conduct the phototropic response experiments a box was constructed of red plexi-glass sheet (8 × 10 × 8 in^3^) to house two light bulbs on opposite sides and camera along with the sporangiophore in its glass vial. Light bulb specifications are: Fiet electric BPEFC40/850/LED/2 DAYLIGHT 220–16-44 E330072, 4.5 W/5000 K/300 lm 120VAC/60 Hz/33 mA. A Sony DSLR-A200 with a Tamron P 90 mm f/2.8 Di Macro Lens camera was used to capture front view images of the sporangiophore to determine change in length (elongation rate) and bending towards the light source (bend angle), while a Celestron 5MP handheld digital microscope captured top view images of the sporangium which has a 1–2 mm long fiber (hair) attached to it. This fiber is used to determine the rotation rate. The duration of each experiment was sixty minutes and the cameras capture an image at every minute. The test sporangiophore (in the vial) was transferred to the prescribed location inside the red box in order to adapt to the experimental conditions and lights. Each experiment begins with both light bulbs turned on for the first ten minutes. The bend angle, rotation angle, and length were measured every minute for the rest of the experiment. This ten-minute interval was used to determine the elongation rate, rotation rate, and bend rate before the phototropic stimulus. At the end of ten-minute interval, one light is turned off. The sporangiophore begins to bend and grow towards the remaining light source. The digital microscope used to capture top view images has a flexible neck and can also be bent along with the sporangiophore to track the rotation rate. At the end of the experiment the images are saved for analysis using MATLAB.

#### Avoidance response experiments

To conduct the avoidance response experiments the red plexi-glass sheet box (8 × 10 × 8 in^3^) with two light bulbs on opposite sides and camera used for the phototropic response experiments were reused. The right side of the box was fitted with a threaded bolt and a cover slip (barrier) was attached to its end. This bolt can be screwed in and out (moving perpendicular to the parallel light from the two light sources), to change the distance between the barrier (cover slip) and growth zone of the sporangiophore. This arrangement orients the surface of the barrier so that it is parallel to the sporangiophore stalk and the light from the light sources on either side, and does not to interfere with the light impinging on the sporangiophore from the side. A Sony DSLR-A200 with a Tamron P 90 mm f/2.8 Di Macro Lens camera was used to capture front view images of the sporangiophore to determine a change in length (elongation) and bending towards the light source (bend angle), while a Celestron 5MP handheld digital microscope is used to capture top view images of the sporangium which has a 1–2 mm long fiber (hair) attached to it. The duration of each experiment was sixty minutes and the cameras capture an image at every minute. The experimental protocol was as follows. Initially, a stage IVb sporangiophore in its vial was placed inside the red box in order to adapt it to the experimental conditions and lights. Each experiment begins with the barrier being at a long distance from the sporangiophore with both lights illuminated. The bend angle, rotation angle and length were measured every minute for the rest of the experiment. A ten-minute interval was used to determine the elongation rate, rotation rate and bend rate before the barrier stimulus was given. At the end of the ten-minute interval, the barrier was moved closer to the sporangiophore using the threaded bolt until a distance of less than a millimeter between the two was reached. Soon after, the sporangiophore begins to bend away from the barrier. The digital microscope used to capture the top view images has a flexible neck and can also be bent along with the sporangiophore to track the rotation rate. At the end of the experiment the images are saved for analysis using MATLAB.

### Stiff mutant experiments

#### Biological material

Vegetative spores of the stiff mutant gene strain C216 *geo -(-)* were originally obtained from Ishinomaki Senshu University, Miyagi, Japan. The C149 *madD120(-)* strain was obtained from ATCC: The Global Research Center, Virginia, USA. Sporangiophores were inoculated on sterile growth medium consisting of 4% (w/v) YM agar. After inoculating, the sporangiophores were incubated under diffuse incandescent light and constant temperature (22 ± 2 °C). Stage IVb sporangiophores, 1.5–2.5 cm in length, were selected for experiments from the second to the seventh crop^21,55^.

#### Elongation rate

The elongation rate of the sporangiophore was determined by measuring the change in length, ∆L, of the bottom edge of the sporangium at 1-min intervals. The length of the sporangium was measured by taking images with a customized camera-microscope set-up made of a web camera (720P HD, GUCEE HD92 Skype Web Camera) attached to a long focal length horizontal microscope (Gaertner;7011Keyepiece and 32 m/m EFL objective) mounted to a 3-D micromanipulator (Line ToolCo.;modelH-2, with digital micrometer heads). The images were acquired using open source software Vividia Ablescope and were analyzed using open source software ImageJ^63^. Prior to initiating photographic measurements, the diameter of the sporangium was measured and later used as a scale reference in ImageJ^21,55^.

#### Rotation rate

**Figure 13:**
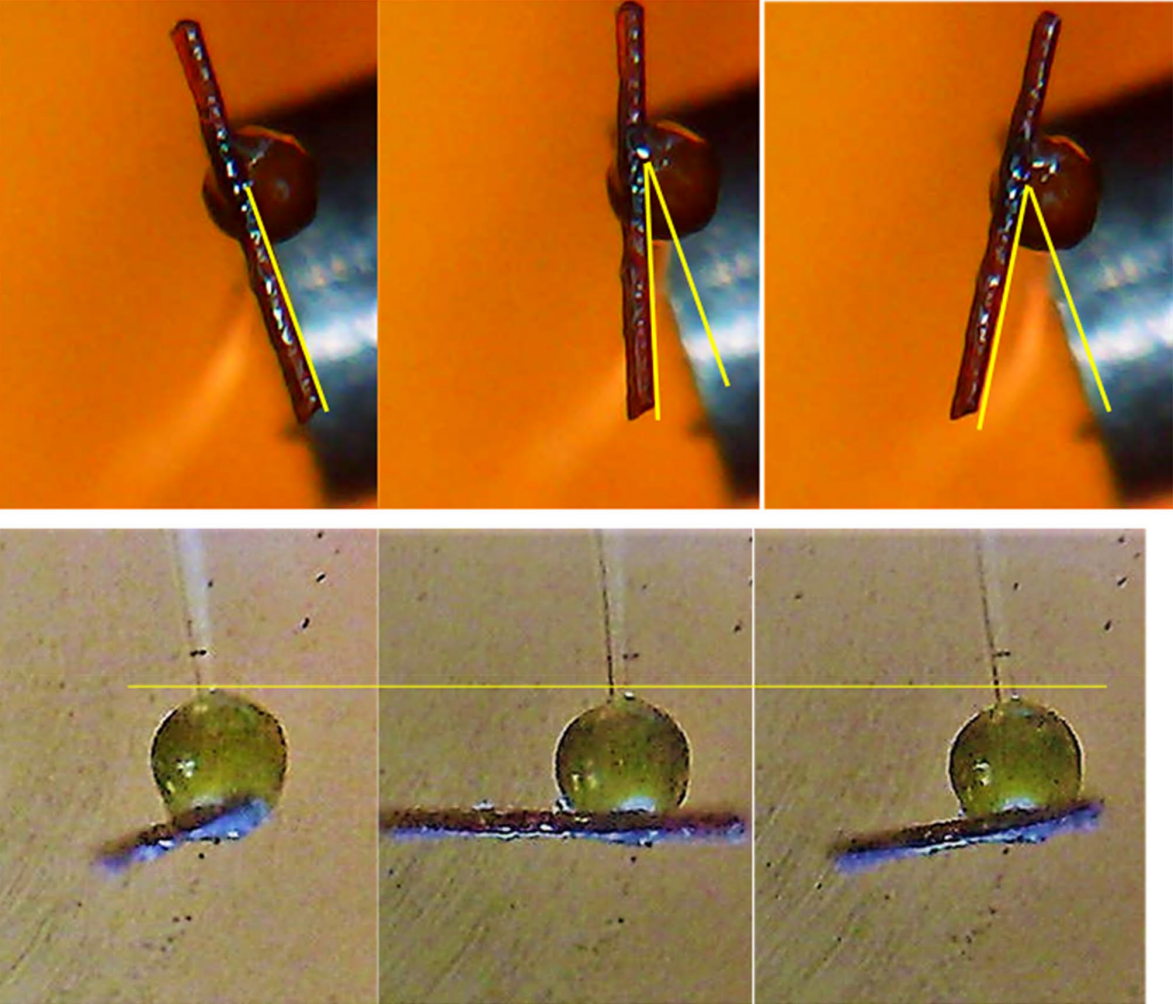
Photographic images showing a short piece of hair attached to the sporangium used to track the sporangium’s rotation rate (top three frames). Photographic images used to determine the elongation rate for each sporangiophore (bottom three frames). The same time interval was used to determine rotation and elongation rate. [Figure was taken from Munoz^55^ and reproduced here with permission].

A short piece of hair (7–10 mm long) was attached to the top of the sporangium of the stage IVb sporangiophore to track the sporangium’s rotation. The hair was placed perpendicular to the longitudinal axis of the cell and secured using a small amount of petroleum jelly. A USB microscope was used to take photographic images of the hair attached to the sporangium and to help determine the rotation rate. The images were acquired using open source software Vividia Ablescope and were analyzed using open source software ImageJ^55,63^. Figure 13 shows snapshots for elongation rate and rotation rate measurements.

## References

[1] Castle ES (1942). Spiral growth and reversal of spiraling in *Phycomyces* and their bearing on primary wall structure. Am. J. Bot..

[2] Bergman K, Burke PV, Cerdá-Olmedo E, David CN, Delbruck M, Foster KW, Goodell EW, Heisenberg M, Meissner G, Zalokar M, Dennison DS, Shropshire W (1969). Phycomyces. Bacteriol. Rev..

[3] Cerda-Olmedo E, Lipson ED (1987). Phycomyces.

[4] Dennison, D.S. Studies on phototropic equilibrium and phototropic-geotropic equilibrium in *Phycomyces*. *Ph.D. Thesis.* 1–146 (California Institute of Technology, 1958).

[5] Ortega JKE, Truong JT, Munoz CM, Ramirez DG (2015). Cell wall loosening in the fungus *Phycomyces blakesleeanus*. Plants.

[6] Elfving F (1881). En obeaktad kanslighet hos *Phycomyces*. Bot Not..

[7] Castle ES (1966). Light responses of *Phycomyces*. Science.

[8] Foster KW, Lipson ED (1973). The light growth response of *Phycomyces*. J Gen Physiol..

[9] Delbruck M, Reichardt W, Rudnick D (1956). System analysis for the light growth reactions of *Phycomyces*. Cellular Mechanisms in Differentiation and Growth.

[10] Ortega JKE, Smith ME, Espinosa MA (1995). Cell wall extension behavior of *Phycomyces* sporangiophores during the pressure response. Biophys. J..

[11] Dennison DS, Roth CC (1967). *Phycomyces* sporangiophores: Fungal stretch receptors. Science.

[12] Castle ES (1933). The physical basis of the positive phototropism of *Phycomyces*. J. Gen. Physiol..

[13] Dennison DS (1965). Steady-state phototropism in *Phycomyces*. J. Gen. Physiol..

[14] Castle ES (1965). Differential growth and phototropic bending in *Phycomyces*. J. Gen. Physiol..

[15] Dennison DS, Foster KW (1977). Intracellular rotation and the phototropic response of *Phycomyces*. Biophys. J..

[16] Gamow RI, Bottger B (1982). *Phycomyces*: Discovery of the aiming error in the avoidance response. Plant Physiol..

[17] Cohen RJ, Jan NY, Matricon J, Delbruck M (1975). Avoidance response, house response and wind responses of the sporangiophore of *Phycomyces*. J. Gen. Physiol..

[18] Ootaki T, Miyazaki A (1993). Genetic nomenclature and strain catalogue of *Phycomyces*.

[19] Campuzano V, Galland P, Alvarez MI, Eslava AP (1996). Blue-light receptor requirement for gravitropism, autochemotropism and ethylene response in *Phycomyces*. Photochem. Photobiol..

[20] Grolig F, Eibel P, Schimek C, Schapat T, Dennison DS, Galland PA (2000). Interaction between gravitropism and phototropism in sporangiophores of *Phycomyces blakesleeanus*. Plant Physiol..

[21] Munoz CM, Ortega JKE (2019). Dimensionless numbers to study cell wall deformation of stiff mutants of *Phycomyces blakesleeanus*. Plant Direct..

[22] Oort AJP (1931). The spiral growth of *Phycomyces*. Proc. K. Ned. Akad. Wet..

[23] Green PB (1954). The spiral growth pattern of the cell wall in *Nitella axillaris*. Am. J. Bot..

[24] Heyn ANJ (1936). Further investigations on the mechanism of cell elongation and the properties of the cell wall in connection with elongation, IV, Investigation on the molecular structure of chitin cell wall of sporangiophores of *Phycomyces* and its probable bearing on the phenomenon of spiral growth. Protoplasma.

[25] Roelofsen PA (1950). The origin of spiral growth in *Phycomyces* sporangiophores. Rec. Trav. Bot. Neer..

[26] Middlebrook MJ, Preston RD (1952). Spiral growth and spiral structure, III, Wall structure in the growth zone of *Phycomyces*. Biochim. Biophys. Acta.

[27] Preston RD (1974). The physical biology of plant cell walls.

[28] Castle ES (1937). The distribution of velocities of elongation and of twist in the growth zone of *Phycomyces* in relation to spiral growth. J. Cell Comp. Physiol..

[29] Cohen R, Delbruck M (1958). Distribution of stretch and twist along the growing zone of the sporangiophore of *Phycomyces* and the distribution of response to a periodic illumination program. J. Cell Comp. Physiol..

[30] Ortega JKE, Harris JF, Gamow RI (1974). The analysis of spiral growth in *Phycomyces* using a novel optical method. Plant Physiol..

[31] Gamow RI, Geer AG, Bottger B (1986). *Phycomyces*: Fine structure analysis of the growing zone. Plant Physiol..

[32] Ortega JKE, Gamow RI (1974). The problem of handedness reversal during the spiral growth of *Phycomyces*. J. Theor. Biol..

[33] Yoshida K, Ootaki T, Ortega JKE (1980). Spiral growth in the radially-expanding piloboloid mutants of *Phycomyces blakesleeanus*. Planta.

[34] Ortega JKE, Lesh-Laurie GE, Espinosa MA, Ortega EL, Manos SM, Cunning MD, Olson JEC (2003). Helical growth of stage-IVb sporangiophores *Phycomyces blakesleeanus*: the relationship between rotation and elongation growth rates. Planta.

[35] Ortega, J.K.E. *Phycomyces*: The mechanical and structural dynamics of cell wall growth. *Ph.D. Thesis.* 1–302 (University of Colorado, Boulder CO, 1976).

[36] Cosgrove DJ (1993). How do plant cell walls extend?. Plant Physiol..

[37] Cosgrove DJ (1993). Wall extensibility: its nature, measurement and relationship to plant cell growth. New Phytol..

[38] Ortega JKE (1985). Augmented growth equation for cell wall expansion. Plant Physiol..

[39] Ortega JKE, Keanini RG, Manica KJ (1988). Pressure probe technique to study transpiration in *Phycomyces* sporangiophores. Plant Physiol..

[40] Ortega JKE (1990). Governing equations for plant cell growth. Physiol Plant..

[41] Ortega JKE (2010). Plant cell growth in tissue. Plant Physiol..

[42] Ortega, J.K.E. A quantitative biophysical perspective of expansive growth for cells with walls. *Rec Res Dev Biophys*. **3** (Part II), 297–324 (2004). ISBN: 81-7895-130-4

[43] Ortega, J.K.E. Growth rate regulation of cells with walls: The sporangiophores of *Phycomyces blakesleeanus* used as a model system. *Rec Res Dev Plant Physiol*. **5**, 1–19 (2012). ISBN: 978-81-308-0497-2

[44] Ortega JKE, Manica KJ, Keanini RG (1988). *Phycomyces*: Turgor pressure behavior during the light and avoidance growth responses. Photobiol. Photochem..

[45] Ortega JKE (2016). Dimensional analysis of expansive growth of cells with walls. Res. Rev. J Bot Sci..

[46] Ortega JKE (2018). Dimensionless numbers for plant biology. T Plant Sci..

[47] Ortega JKE (2019). Dimensionless numbers to analyze expansive growth processes. Plants..

[48] Ortega JKE (2017). Dimensionless number is central to stress relaxation and expansive growth of the cell wall. Sci. Rep..

[49] Sridhar SL, Ortega JKE, Vernerey FJ (2018). A statistical model of expansive growth in plant and fungal cells: The case of *Phycomyces*. Biophys. J..

[50] Benet E, Zhu H, Vernerey FJ (2019). Interplay of elastic instabilities and viscoelasticity in the finite deformation of thin membranes. Phys. Rev. E..

[51] Vernerey FJ, Long R, Brighenti R (2017). A statistically-based continuum theory for polymers with transient networks. J. Mech. Phys. Solids..

[52] Vernerey FJ (2018). Transient response of nonlinear polymer networks: A kinetic theory. J. Mech. Phys. Solids..

[53] Lalitha Sridhar S, Vernerey FJ (2018). The chain distribution tensor: linking nonlinear rheology and chain anisotropy in transient polymers. Polymers.

[54] Lalitha Sridhar S, Vernerey FJ (2020). Mechanics of transiently cross-linked nematic networks. J. Mech. Phys. Solids.

[55] Munoz, C.M. Dimensional analyses to quantify cell wall deformation and stress relaxation in stiff mutants of *Phycomyces blakesleeanus*: Experimental investigations. *Ph.D. Thesis.* 1–114 (University of Colorado, Denver, CO, 2018).

[56] Ortega JKE, Munoz CM, Blakley SE, Truong JT, Ortega EL (2012). Stiff mutant genes of *Phycomyces* affect turgor pressure and wall mechanical properties to regulate elongation growth rate. Front Plant Sci..

[57] Ahlquist CN, Gamow RI (1973). *Phycomyces*: Mechanical behavior of stage II and stage IV. Plant Physiol..

[58] Roelofsen PA (1951). Cell wall structure in the growth zone of Phycomyces sporangiophores. II. Double refraction and electron microscopy. Biochim. Biophys. Acta.

[59] Trinci APJ, Halford EA (1975). The extension zone of stage I sporangiophores of *Phycomyces blakesleeanus*. New Phytol..

[60] Ortega JKE, Zehr EG, Keanini RG (1989). In vivo creep and stress relaxation experiments to determine the wall extensibility and yield threshold for the sporangiophores of *Phycomyces*. Biophys. J..

[61] Jan, Y.N. (I) Chitin synthetase and sensory transduction process in *Phycomyces*, (II) The avoidance response, the house growth response, and the rheotropic response of *Phycomyces*. *Ph.D. Thesis*. (California Institute of Technology, Pasadena, California, 1974).

[62] Ruiz-Herrera J (2016). Fungal Cell Wall: Structure, Synthesis, and Assembly.

[63] Rasband, W.S., ImageJ, U. S. National Institutes of Health, Bethesda, Maryland, USA, https://imagej.nih.gov/ij/, 1997–2018.

